# Correlation of ACR and TcPO2 in diabetic kidney disease patients: A pilot study

**DOI:** 10.1111/1753-0407.13385

**Published:** 2023-04-25

**Authors:** Jin Sun, Yang Huang, Lanhua Li, Hao Hu, Yuanyuan Liu, Xuelian Zhang, Hao Zhang, Binbin Pan

**Affiliations:** ^1^ Department of Endocrinology Xuzhou First People's Hospital, The Affiliated Xuzhou Municipal Hospital of Xuzhou Medical University Xuzhou People's Republic of China; ^2^ Department of Gerontology, Nanjing First Hospital Nanjing Medical University Nanjing People's Republic of China; ^3^ Department of Nuclear Medicine, Nanjing First Hospital Nanjing Medical University Nanjing People's Republic of China; ^4^ Department of Nephrology, Nanjing First Hospital Nanjing Medical University Nanjing People's Republic of China

**Keywords:** diabetic kidney disease, microcirculation, ratio of albuminuria and creatinine, transcutaneous oxygen pressure, 经皮氧分压, 尿白蛋白与肌酐比值, 糖尿病肾病, 微循环

## Abstract

**Objective:**

Transcutaneous oxygen pressure (TcPO2) is used to assess microcirculation clinically; however, it is not widely available especially in rural hospital. The study was designed to explore potential alternatively biomarkers to assess microcirculation in diabetic kidney disease (DKD).

**Methods:**

A total of 404 patients from Xuzhou first hospital were recruited according to the case records system. Patients were grouped via the ratio of albuminuria and creatinine (ACR; <30 mg/g, 30−300 mg/g, >300 mg/g). Biomarkers in different ACR groups were compared by analysis of variance. Correlation analysis was determined by Pearson or Spearman analysis and binary logistic regression. The receiver operating characteristics (ROC) curve was performed to elucidate the prediction effect of ACR on TcPO2.

**Results:**

A total of 404 diabetic patients were recruited with 248 patients diagnosed as DKD and 156 non‐DKDs. Age and cystatin C were significantly higher in the ACR3 group compared with those in the ACR1 group, whereas glomerular filtration rate, low‐density lipoprotein cholesterol, and TcPO2 were markedly decreased in the ACR3 group (*p* < .05). Frequency of low TcPO2 (<40 mm Hg) was markedly increased as increment of ACR stages with 30.2% in the ACR3 group (*p* < .01). There was a negative correlation between TcPO2 and age, ACR, chronic kidney disease (CKD), fast blood glucose, diabetes mellitus (DM) duration, and diabetic neuropathy. Further, binary logistic regression showed ACR was an independent influence factor for low TcPO2. After adjusting for age, gender, hypertension, DM duration, body mass index, glycated hemoglobin, diabetic neuropathy, and CKD, ACR was still an independent influence factor for TcPO2 (odds ratio = 2.464, *p* < .01). The area under the ROC curve was 0.768 (95% confidence interval: 0.700–0.836, *p* < .001) for ACR. The analysis of ROC curves revealed a best cutoff for ACR was 75.25 mg/g and yielded a sensitivity of 71.7% and a specificity of 71.7%.

**Conclusions:**

ACR could be used as an alternative biomarker for assessing microcirculation in DKD patients.

## Graphical abstract

**Highlights**

- Transcutaneous oxygen pressure (TcPO2) could be used to assess microcirculation, which revealed endothelial injury. As endothelial injury is a cause of albuminuria in diabetic kidney disease (DKD), albuminuria and creatinine (ACR) might be employed to assess microcirculation in DKD patients in rural hospital where TcPO2 is not available. Our study showed TcPO2 was significantly lower in patients with ACR >300 mg/g and a negative correlation between TcPO2 and ACR was observed. Further, ACR was an independent influence factor of low TcPO2. On top of that, the area under receiver operating characteristics curve was 0.768 for ACR in predicting TcPO2. The best cutoff value for ACR was 75.25 mg/g and yielded a sensitivity of 71.7% and a specificity of 71.7%. These results suggested ACR could be used as a potential biomarker for assessing microcirculation in DKD patients.

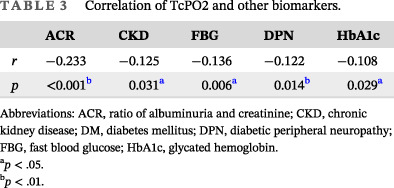

## 摘要

**【摘要】 目的** 经皮氧分压(TcPO2)被用于临床评估微循环，但在农村医院应用并不广泛，因此本研究旨在探索潜在的替代性生物标志物，以评估糖尿病肾病(DKD)患者的微循环情况。**方法** 根据病案记录系统，共招募了徐州市第一医院的404例患者。根据尿白蛋白与肌酐比值(ACR)，将患者分为3组(<30 mg/g，30~300 mg/g，>300 mg/g)。采用方差分析比较不同ACR组中的生物标志物。通过*Pearson*或*Spearman*相关分析和二元*logistic*回归进行相关性分析。利用ROC曲线来解释ACR对TcPO2的预测效果。**结果** 共招募了404例糖尿病患者，其中248例诊断为DKD，156例为非DKD。与ACR1组相比，ACR3组的年龄和胱抑素C显著升高，而GFR、LDL和TcPO2明显降低(*P*<0.05)。低TcPO2[<40 mmHg(1 mmHg=0.133 kPa)]的发生率随着ACR分组的增加而显著增加，ACR3组为30.2%(*P*<0.01)。TcPO2与年龄、ACR、CKD、空腹血糖、糖尿病病程和糖尿病神经病变之间存在负相关关系。进一步的二元*logistic*回归分析显示，ACR是低TcPO2的独立影响因素。在校正了年龄、性别、高血压、糖尿病病程、BMI、HbA_1C_、糖尿病神经病变和CKD等因素后，ACR仍然是TcPO2的独立影响因素(*OR*=2.464，*P*<0.01)。ACR的ROC曲线下面积为0.768(95% *CI* 0.700~0.836，*P*<0.001)。ROC曲线分析显示，ACR的最佳截断点为75.25 mg/g，敏感性为71.7%，特异性为71.7%。**结论** ACR可作为评估DKD患者微循环的替代生物标志物。

## 1 INTRODUCTION

Type 2 diabetes is prevalent all over the world with 11.2% of prevalence in China.^1^ With hyperglycemia and fluctuation of blood glucose, diabetic complications develop that influence life quality and impose a big economic burden. Managing blood glucose and diabetic complications could slow down progression of complications and prevent severe cardiovascular events. Microvascular structure, also known as microcirculation, controls the bloodstream of heart, kidney, retina, skin, and other organs. Disorder of microcirculation is associated with increased platelet reactivity, decreased vasodilator responses and fibrinolysis, and remodeling and rarefaction of microvessels, resulting in development and progression of hypertension, chronic kidney disease (CKD), diabetes, and cardiovascular disease.^2^ Decline of microcirculation exhibits low‐density of retinal vessels, retinal arteriolar narrowing,^3^ wider venules,^4^ and reduced fractal dimension by retinal optical coherence tomography.

Transcutaneous oxygen pressure (TcPO2) is frequently used as a metric to assess microcirculation in diabetes, especially in diabetic foot ulcer patients. TcPO2 <25 mm Hg could be used to predict increased 1‐year mortality in diabetic foot ulcer patients.^5^ TcPO2 was also used to monitor the treatment effect of autologous cell therapy and the result showed that a higher TcPO2 level after receiving cell therapy was associated with a significantly longer amputation‐free survival time.^6^ Dialysis could influence microcirculation at least 4 h manifesting as decreased TcPO2.^7^, ^8^ In diabetic retinopathy patients, low TcPO2 (<30 mm Hg) occurred in 18.6% patients compared with 6.26% in nondiabetic patients revealing decline of microcirculation in these patients.^9^

Previous study also proved TcPO2 could predict diabetic complications, including diabetic kidney disease (DKD). However, no data to date are available about the correlation of TcPO2 and DKD biomarkers. As is known, DKD is also associated with disorders of microcirculation, which is essential to be assessed in DKD patients. Nevertheless, TcPO2 is not widely used, especially in rural hospitals. It is important to explore alternative biomarkers to assess microcirculation in DKD.

## 2 MATERIALS AND METHODS

A total of 404 patients were recruited from January 2018 to August 2019 in Xuzhou First People's Hospital. We excluded subjects as follows: younger than 18 years old, heart failure, fever, liver dysfunction, pregnancy, nonketotic hyperosmolar coma, diabetic lactic acidosis, diabetic ketoacidosis, and acute kidney injury.

### 2.1 Laboratory measure

Cystatin C, serum creatinine (sCr), uric acid, β2­microglobin, serum albumin, calcium, phosphorus, serum lipid, and proteinuria were determined by Beckmann AU5800 automatic biochemical analyzer. Ratio of albuminuria and creatinine (ACR) was determined via immunoturbidimetry. Glycated hemoglobin (HbA1c) was determined by HPLC (BIO‐RAD). Homocysteine (HCY)was detected by enzymatic cycling assay.

### 2.2 Definition of CKD

Different CKD stages were divided according to estimated glomerular filtration rate (eGFR) following Kidney Disease: Improving Global Outcomes (KDIGO) guideline^10^: stage 1, eGFR ≥90 mL/min/1.73m^2^; stage 2, 60 mL/min/1.73m^2^ ≤ eGFR <90 mL/min/1.73m^2^; stage 3, 30 mL/min/1.73m^2^ ≤ eGFR <60 mL/min/1.73m^2^; stage 4, 15 mL/min/1.73m^2^ ≤ eGFR <30 mL/min/1.73m^2^; stage 5, and eGFR <15 mL/min/1.73m^2^. Chronic Kidney Disease Epidemiology Collaboration (CKD­EPI) four­level race equation was employed to calculate eGFR. The specific CKD­EPI four­level race GFR estimation equation was as follows,

$$\text{eGFR}=\begin{array}{l} \mathrm{EXP}(\mathrm{LN}\left(151\right)-0.328\times \mathrm{LN}\left(\mathrm{sCr}/88.4/0.7\right)+\mathrm{age}\\ \times \mathrm{LN}\left(0.993\right))\left(\text{If female and}\;\mathrm{sCr}<0.7\right); \end{array}$$

$$=\begin{array}{l} \mathrm{EXP}(\mathrm{LN}\left(151\right)-1.210\times \mathrm{LN}\left(\mathrm{sCr}/88.4/0.7\right)+\mathrm{age}\\ \times \mathrm{LN}\left(0.993\right))\left(\text{If female and}\;\mathrm{sCr}\geq 0.7\right); \end{array}$$

$$=\begin{array}{l} \mathrm{EXP}(\mathrm{LN}\left(149\right)-0.412\times \mathrm{LN}\left(\mathrm{sCr}/88.4/0.9\right)+\mathrm{age}\\ \times \mathrm{LN}\left(0.993\right))\left(\text{If male and}\;\mathrm{sCr}<0.9\right); \end{array}$$

$$=\mathrm{EXP}\left(\mathrm{LN}\left(149\right)-1.210\times \mathrm{LN}\left(\mathrm{sCr}/88.4/0.9\right)+\mathrm{age}\times \mathrm{LN}\left(0.993\right)\right)\;\left(\text{If male and}\;\mathrm{sCr}\geq 0.9\right).$$

### 2.3 ACR groups definition

ACR groups were defined as follows: ACR1 group, ACR <30 mg/g; ACR2 group, 30 mg/g ≤ ACR ≤ 300 mg/g; and ACR3 group, ACR > 300 mg/g.

### 2.4 Diabetic peripheral neuropathy (DPN) definition

DPN was diagnosed according to the American Diabetes Association guideline of 2010.^11^ Patients with other etiological neuropathy, such as metabolic, hereditary, cervical, and lumbar spine diseases, inflammatory issues; uremia; alcohol use; cerebrovascular diseases; and toxic factors, were excluded.

#### 2.4.1 TcPO2 determination

TcPO2 measurements were performed at the dorsum of both feet. The patients position was supine. The determination of TcPO2 should not be performed in edema feet. If the patients manifested edema feet, edema should be improved before determination.

### 2.5 Statistical analysis

Statistical analysis was performed by PASW 22.0 statistical software (SPSS Inc., Chicago, IL, USA). Data were expressed as mean ± SD. One‐way analysis of variance was used to compare means for continuous variables. The least significant difference method was used for continuous variables with homogeneous variances and Dunnett's method was used for continuous data with uneven variances. The Pearson or Spearman correlation analysis was employed to determine the correlations between TcPO2 and other indices. In addition, the binary logistic regression was employed to explore independent influence factors for TcPO2. Receiver operating characteristic (ROC) curve was built to evaluate the predicting ability of ACR on TcPO2. The best cutoff for the ROC curve was calculated with the Youden's index. *p* value <.05 was considered to be statistically significant.

## 3 RESULTS

### 3.1 Characteristics of clinical indices among different ACR groups

A total of 404 diabetic patients were recruited with 248 patients diagnosed as DKD and 156 patients without DKD. Age and cystatin C were significantly higher in the ACR3 group compared with those in the ACR1 group, whereas GFR, low‐density lipoprotein (LDL), and TcPO2 were markedly decreased in ACR3 group (*p* <.05). A lower level of hemoglobin was revealed in the ACR3 group (129.74 ± 18.0 g/L) compared with that in the ACR1 and ACR2 groups (*p* < .05) whereas no significant difference was observed between the ACR1 (139.16 ± 16.1 g/L) and ACR2 groups (135.12 ± 16.0) (*p* > .05). Levels of albumin were decreased as increments of ACR levels with the lowest in the ACR3 group (37.56 ± 4.84 g/L). Diabetes mellitus (DM) duration in the ACR2 and ACR3 groups were remarkably higher than that in the ACR1 group. No significant differences were observed in gender, body mass index (BMI), HbA1c, uric acid, serum creatinine, total cholesterol, triglyceride, high density lipoprotein, HCY, and C‐peptide among different groups (Table 1).

**TABLE 1:** Characteristics of clinical indices among different albuminuria and creatinine (ACR) groups.

	ACR1 (<30) 156	ACR2 (30–300) 205	ACR3 (>300) 43
Age	55.71 ± 10.3	59.58 ± 12.2	61.72 ± 13.44^b^
Gender (male/female)	100/56	111/94	25/18
BMI	25.01 ± 2.72	25.17 ± 3.52	25.76 ± 4.28
HbA1c	8.85 ± 2.32	9.23 ± 2.17	10.04 ± 2.43^a^ ^,^ ^b^
Hemoglobin	139.16 ± 16.1	135.12 ± 16.0	129.74 ± 18.0^a^
Albumin	42.73 ± 3.69	41.17 ± 3.66^c^	37.56 ± 4.84^a^ ^,^ ^b^
Cystatin C	0.75 ± 0.16	0.68 ± 0.21^c^	0.79 ± 0.24^b^
Albumin	42.73 ± 3.69	41.17 ± 3.66	37.56 ± 4.84^b^
Uric acid	300.7 ± 80.6	268.4 ± 75.6^c^	292.9 ± 89.9
Serum creatinine	53.14 ± 11.49	53.75 ± 16.24	70.71 ± 67.84
GFR	128.3 ± 30.0	104.2 ± 19.1^c^	96.2 ± 28.4^a^
Tc	5.03 ± 1.51	4.95 ± 1.29	4.62 ± 1.15
Tg	2.09 ± 2.13	1.94 ± 2.41	1.92 ± 1.33
HDL	1.24 ± 0.31	1.29 ± 0.36	1.27 ± 0.37
LDL	3.04 ± 0.82	2.88 ± 0.86	2.48 ± 0.80^a^ ^,^ ^b^
HCY	10.25 ± 5.98	9.87 ± 8.84	9.94 ± 5.33
C‐peptide	3.11 ± 2.74	2.75 ± 1.84	2.96 ± 1.54
TcPO2	56.6 ± 9.4	53.5 ± 11.7^c^	47.9 ± 13.1^a^ ^,^ ^b^
DM duration	78.45 ± 92.80	129.16 ± 93.71^c^	122.21 ± 97.08^a^

Abbreviations: ACR, ratio of albuminuria and creatinine; BMI, body mass index; DM, diabetic mellitus; GFR, glomerular filtration rate; HbA1c, glycated hemoglobin; HCY, homocysteine; HDL, high‐density cholesterol; LDL, low‐density cholesterol; Tc, total cholesterol; Tg, triglyceride; TcPO2, transcutaneous pressure oxygen.

^a^ *p* < .05 (ACR3 vs ACR1).

^b^ *p* < .05 (ACR3 vs ACR2).

^c^ *p* < .05 (ACR2 vs ACR1).

Incidences of diabetic neuropathy and hypertension reveal no siginficant differences among the three groups. Frequency of low TcPO2 (<40 mm Hg) was markedly increased as increment of ACR stages with 30.2% in the ACR3 group (*p* < .01) (Table 2).

**TABLE 2:** Incidences of diabetic neuropathy and hypertention.

	ACR1 (<30)	ACR2 (30–300)	ACR3 (>300)	Chi‐square	*p* value
Low TcPO2	4 (2.6%)	29 (14.1%)	13 (30.2%)	12.841	.001^a^
DPN	94 (60.3%)	113 (55.1%)	26 (60.5%)	1.11	.574
Hypertension	63 (40.4%)	108 (53.2%)	19 (45.2%)	5.889	.052

Abbreviations: ACR, ratio of albuminuria and creatinine; DPN, diabetic peripheral neuropathy; TcPO2, transcutaneous pressure oxygen.

^a^ *p* < 0.01.

### 3.2 Correlations of TcPO2 and clinical indices

There was a negative correlation between TcPO2 and ACR, CKD, fast blood glucose, diabetic neuropathy, and HbA1c (*r* = −0.233, *p* < .01; *r* = −0.125, *p* = .031; *r* = −0.136, *p* < .006; *r* = −0.122, *p* = .014; *r* = −0.108, *p* = .029) (Table 3).

**TABLE 3:** Correlation of TcPO2 and other biomarkers.

	ACR	CKD	FBG	DPN	HbA1c
*r*	−0.233	−0.125	−0.136	−0.122	−0.108
*p*	<0.001^b^	0.031^a^	0.006^a^	0.014^b^	0.029^a^

Abbreviations: ACR, ratio of albuminuria and creatinine; CKD, chronic kidney disease; DM, diabetes mellitus; DPN, diabetic peripheral neuropathy; FBG, fast blood glucose; HbA1c, glycated hemoglobin.

^a^ *p* < .05.

^b^ *p* < .01.

Further, binary logistic regression was performed with low TcPO2 or not as dependent variances. As ACR was not a normal distribution, it was transferred as log_10_ACR. The result showed that ACR was an independent influence factor for low TcPO2. After adjusting for age, gender, hypertension, DM duration, BMI, HbA1c, diabetic neuropathy, and CKD, ACR was still an independent influence factor for TcPO2 (odds ratio [OR] = 2.464, *p* < .01) (Table 4).

**TABLE 4:** Logistic regression analysis using TcPO2 as the dependent variable.

	OR	95% CI	*p* value
Male sex	0.719	0.36–1.435	.349
Age	1.006	0.972–1.04	.748
BMI	1.030	0.935–1.133	.55
HbA1c	1.101	0.939–1.292	.236
DPN	0.860	0.415–1.783	.685
DM duration	1.004	1.001–1.008	.026
Hypertension	0.853	0.403–1.805	.677
ACR	2.464	1.132–5.361	.023^a^
CKD	0.691	0.163–4.693	.654

Abbreviations: ACR, ratio of albuminuria and creatinine; BMI, body mass index; CI, confidence interval; CKD, chronic kidney disease; DM, diabetic mellitus; DPN, diabetic peripheral neuropathy; HbA1c, glycated hemoglobin; OR, odds ratio.

^a^ *p* < .05.

### 3.3 Medication administration in different ACR groups

Medication administration in different ACR groups was collected, including statins, angiotensin‐converting enzyme inhibitor/angiotensin receptor blockers, sulfonylurea, metformin, glucosidase inhibitor, glinides, sodium‐glucose cotransporter‐2 inhibitor, glucagon‐like peptide‐1, and dipeptidyl peptidase‐4 inhibitor. No significant differences were observed among ACR groups in frequency of different medications (*p* > .05) (Table 5).

**TABLE 5:** Medications administrations in different ACR groups.

Medications	ACR1	ACR2	ACR3	Chi‐square	*p* value
Statins	9 (5.8%)	27 (13.2%)	5 (11.6%)	5.438	.066
ACEI	4 (2.6%)	13 (6.3%)	1 (2.3%)	3.482	.175
ARB	21 (13.5%)	37 (18%)	3 (7%)	3.931	.14
Sulfonylurea	30 (19.2%)	46 (22.4%)	13 (30.2%)	2.416	.299
Metformin	81 (51.9%)	115 (51.6%)	22 (51.2%)	0.773	.679
Glucosidase inhibitor	33 (21.2%)	57 (27.9%)	11 (25.6%)	2.175	.337
Glinides	15 (9.6%)	30 (14.6%)	2 (4.7%)	4.453	.108
SGLT2 inhibitor	12 (7.7%)	25 (12.2%)	1 (2.3%)	4.939	.085
GLP‐1	5 (3.2%)	12 (5.9%)	0 (0%)	3.656	.161
DPP‐IV inhibitor	2 (1.3%)	2 (1%)	0 (0%)	0.566	.753

Abbreviations: ACEI, angiotensin‐converting enzyme inhibitor; ACR, ratio of albuminuria and creatinine; ARB, angiotensin receptor blockers; DPP‐IV, dipeptidyl peptidase‐4; GLP‐1, glucagon‐like peptide‐1; SGLT2, sodium‐glucose cotransporter‐2.

### 3.4 The efficiency of ACR to predict TcPO2

**FIGURE 1:**
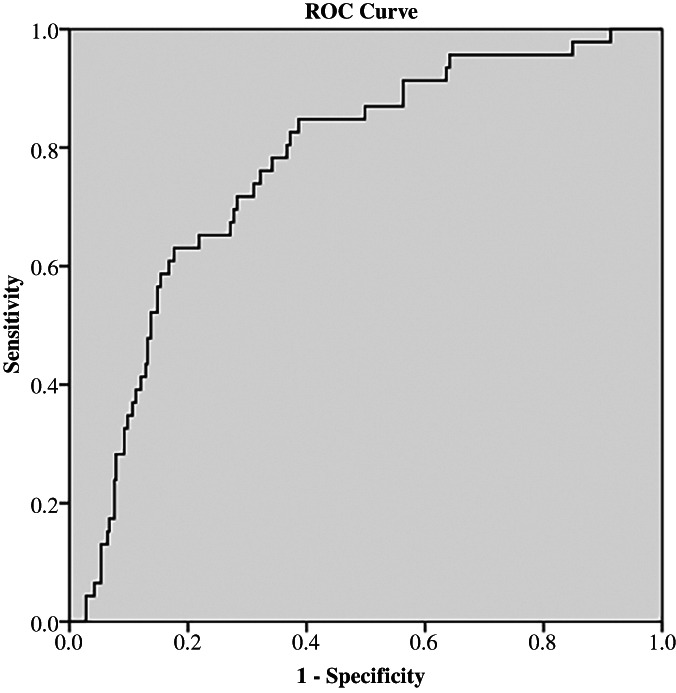
Receiver operating characteristic (ROC) curves for albuminuria and creatinine (ACR) for prediction of TcPO2. The area under the ROC was 0.768 (95% confidence interval: 0.700–0.836, *p* < .001) for ACR. The analysis of ROC revealed that the optimal cutoff for ACR was 75.25 mg/g and provided a sensitivity of 71.7% and a specificity of 71.7%. TcPO2, transcutaneous pressure oxygen.

ROC curve was determined to evaluate the prediction efficiency of ACR on low TcPO2 (<40 mm Hg). The area under the ROC curve was 0.768 (95% confidence interval [CI]: 0.700–0.836, *p* < 0.001) for ACR. The analysis of ROC curves revealed a best cutoff for ACR was 75.27 mg/g and yielded a sensitivity of 71.7% and a specificity of 71.7% (Figure 1).

## 4 DISCUSSION

In our study, we investigated the relationship of TcPO2 and ACR. The levels of TcPO2 were decreased accompanied with increased stages of ACR. Further, Pearson analysis revealed a negative correlation between TcPO2 and ACR. Moreover, binary logistic regression showed significant OR value for ACR in influencing levels of TcPO2 after adjusting other clinical indices. On top of that, we performed ROC curve to observe the predicting ability of ACR on TcPO2 and the result exhibited a sensitivity of 71.7% and a specificity of 71.7% suggesting ACR was a potential biomarker for assessing microcirculation in DKD patients.

DM could induce many complications including DKD, DPN, diabetic retinopathy, coronary artery disease, artery plaque, and disorders of microcirculation. Also, microcirculation was correlated with DPN and diabetic ulcers in the foot. DKD also manifested as dysfunction of microcirculation. Capillary density was significantly lower in CKD3b or higher stage patients with diabetes.^12^ Optical coherence tomography angiography revealed decreased retinal vessel density, which was positively correlated with eGFR and ACR was also associated with increased macular thickness.^13^ CKD patients revealed decreased superficial vascular plexus and deep vascular plexus compared with healthy controls.^14^ Perfusion index representing the level of circulation through peripheral tissues was independently correlated with ACR and eGFR. High systolic blood pressure or low perfusion index had markedly increased odds of ACR and eGFR.^15^ Clinically, TcPO2 was also frequently used as a useful tool to assess microcirculation in diabetic patients. Previous study indicated optimal cutoff point (19.5 mm Hg) yielded a sensitivity of 0.611 and a specificity of 0.738 for predicting DPN.^16^ Further, correlations analysis of TcPO2 and diabetic microvascular complications (DKD and/or DPN) showed cutoff value (50 mm Hg) could yield a sensitivity 0.766 and a specificity 0.702.^17^ Our study also indicated negative correlation of TcPO2 and DPN. In diabetic retinopathy patients, the frequency of TcPO2 < 40 mm Hg was 30.23%.^9^ The previous studies indicated that TcPO2 could be a potential index to assess diabetic microvascular complications. On top of that, TcPO2 was also determined as an essential index to assess macrovascular complications, such as diabetic foot ulcer.^18^ Different studies have exhibited variant criterion for low TcPO2. In a diabetic foot ulcer study, < 30 mm Hg was defined as low TcPO2^19^ whereas using TcPO2 to predict survival in patients with diabetic foot ulcer, <25 mm Hg was determined as a significantly low TcPO2.^5^ In predicting major adverse cardiovascular events, <46 mm Hg was set as low TcPO2.^20^ However, <40 mm Hg was defined as low TcPO2 in most studies,^21^, ^22^ which was also the definition in our study. As diabetic kidney disease could induce edema, it may influence the measurement of TcPO2. Although previous studies^23^, ^24^ revealed that treatment of edema might not influence levels of TcPO2, we still measure TcPO2 in patients without lower limb edema.

Although TcPO2 is used to assess microcirculation in DKD, it is not widely available in most hospitals. Therefore, exploring other clinical indices to predict microcirculation in DKD could provide a novel method. However, there is a paucity of studies analyzing correlations of TcPO2 and kidney biomarkers. As most patients in our study were within normal range of kidney function, it is limited to analyze characteristics of TcPO2 in different CKD stages. Also, we performed correlation analysis of TcPO2 and eGFR, which resulted in no significance.

Microcirculation disorder consists of microvascular dysfunction and neuropathy. Endothelial injury could induce accumulation of platelets and develop vessel plaque resulting in decreased bloodstream speed or stasis. Hence, endothelial injury is an important contributing factor for dysfunction of microvessels. Meanwhile, decreased endothelial cells in DKD pathology are common, which is also the cause of micoalbuminuria. In fact, ACR could be used as an biomarker to assess endothelial injury in DKD. Further, we speculated ACR could be used to assess microcirculation in DKD as TcPO2 was negatively correlated with ACR. After dividing participants into different ACR groups, we observed decreased trends as increments in ACR stages. To determine whether ACR could be used as a surrogate biomarkers to assess microcirculation in diabetes, we performed binary logistic regression analysis and ACR was an independent risk factor for low TcPO2, which was further verified after adjusting other clinical indices. Further, ROC curve was performed and the result revealed ACR had a good predicting ability for TcPO2.

Our study also revealed inversely correlation between TcPO2 and fasting blood glucose or age, which was similar to a previous study.^25^ Our study showed TcPO2 was not correlated with DM duration, which had a negative correlation with TcPO2 in our study. Clincally, frequency of diabetic complications is increased with increments of DM duration resulting in deteriorative microcirculation. Furthermore, we performed binary logistic regression and found DM duration was an independent risk factor for low TcPO2 suggesting DM duration was inversely correlated with TcPO2 in multiple factors analysis.

There are some limitations in our study. First, we used TcPO2 as an index of microcirculation, which may not completely reveal its real level. However, we still lack a golden index to assess microcirculation whereas TcPO2 is used most frequently clinically. Second, this was a single‐center study and the sample size was not large enough; further study is needed to verify its specificity and sensitivity.

Taken together, ACR had a negative correlation with TcPO2 and could be identified as a potential biomarker for assessing microcirculaion in DKD patients especially in hospitals where TcPO2 cannot be measured.

## AUTHOR CONTRIBUTIONS

The research was designed by Binbin Pan and Jin Sun. All authors helped to write the report and commented on the manuscript. Jin Sun, Yang Huang, and Lanhua Li analyzed the data and advised on statistical issues at the time of the research write‐up. Binbin Pan and Hao Zhang was the research administrator. Jin Sun, Hao Hu, Yuanyuan Liu, and Xuelian Zhang obtained the data and prepared communications with participating centers and the data monitoring committee.

## FUNDING INFORMATION

This work was supported by grants from the Foundation of Science and Technology Development Program, Nanjing Medical University (NMUB20210214). The funders had no role in study design, data collection and analysis, decision to publish, or reparation of the manuscript.

## CONFLICT OF INTEREST STATEMENT

The authors have nothing to disclose.

## Data Availability

Data are available on request to Dr. Pan (Email: panbinbin@njmu.edu.cn).
